# Associations between social support and central components of adolescent materialism: a network analysis

**DOI:** 10.3389/fpsyg.2026.1925963

**Published:** 2026-10-14

**Authors:** Sen Li, Ruosi Gao, Xiang Li, Tianran Li

**Affiliations:** 1 School of Education, Hebei University, Baoding, China; 2 School of Education, Beijing City University, Beijing, China

**Keywords:** adolescents, materialism, network analysis, social support, teacher support

## Abstract

**Introduction:**

Adolescent materialism has been increasingly associated with a range of psychological health issues. This study explored the central components of materialism and their associations with social support using an undirected partial-correlation network.

**Methods:**

A convenience sample of 1,976 adolescents was recruited from one junior high school and one senior high school in Hebei Province China, and materialism and social support were assessed using previously developed self-report measures. The final analytic sample comprised 1,639 adolescents, with social support assessed at Time 1 and materialism assessed 7 months later at Time 2.

**Results:**

Network analysis revealed complex interrelationships between different sources of social support and distinct facets of materialism. Notably, MVS12 (“I’d be happier if I could afford to buy more things”) showed the highest expected influence, highlighting its central position among the materialism nodes within the estimated network. Furthermore, teacher support showed the strongest negative source-level bridge connectivity with materialistic values.

**Discussion:**

These findings highlight teacher support as a potentially important correlate of adolescent materialism and provide a basis for future longitudinal and intervention research.

## 1 Introduction

Materialism, defined as the emphasis on material possessions as central to individual satisfaction and life goals (Belk, 1985; Richins and Dawson, 1992), has become increasingly pervasive worldwide (Bartolini and Sarracino, 2017). Over the past two decades, materialistic values among adolescents have risen significantly, alongside increasing market and economic globalization (Vinayak and Arora, 2018). Adolescence, a critical transitional phase marked by rapid physiological changes and heightened psychological sensitivity, renders individuals particularly vulnerable to the influence of social values (Blakemore, 2018). This vulnerability may facilitate the internalization of materialistic orientations, which are linked to numerous negative personal outcomes, such as reduced subjective well-being, diminished prosocial behavior, increased aggression, impaired motivation for learning, lower academic achievement, and decreased engagement with school (Dittmar and Isham, 2022; Lv et al., 2023; Wang et al., 2017). Beyond individual consequences, materialism also gives rise to broader societal challenges, including strained parent–child relationships and exacerbated family conflicts (Burroughs and Rindfleisch, 2002), as well as increased social inequality and a diminished sense of morality (Piff et al., 2010, 2012). Moreover, materialism is negatively associated with environmentally sustainable attitudes and behaviors, as adolescents with higher materialistic values tend to exhibit lower levels of environmental concern, potentially exacerbating future ecological issues (Hurst et al., 2013; Kilbourne and Pickett, 2008). Considering the widespread presence and detrimental effects of materialism, a thorough investigation of the factors associated with it is important for informing future prevention research.

Research underscores the close association between social support and adolescent materialism, with the quality of support being related to materialistic values (Chaplin and John, 2010). According to the compensatory consumer behavior model (Mandel et al., 2017), self-discrepancies, referring to perceived gaps between one’s actual and desired self, can generate psychological discomfort and threaten self-worth. In response, individuals may turn to possessions and consumption as symbolic means of affirming a valued identity, gaining social recognition, or restoring a sense of competence and self-worth. When material possessions are repeatedly used to compensate for such insecurity, they may become increasingly central to how individuals define themselves and pursue happiness, thereby contributing to stronger materialistic values. A complementary explanation is provided by self-determination theory (Ryan and Deci, 2000), which proposes that social support can facilitate the satisfaction of adolescents’ basic psychological needs for autonomy, competence, and relatedness. When these fundamental needs are satisfied through supportive interactions, adolescents are less compelled to seek validation through material possessions. Supportive relationships provide emotional acceptance, positive interactions, and a sense of social belonging, which may be associated with lower materialistic tendencies (Chaplin and John, 2010). Conversely, insufficient social support has been associated with stronger materialistic orientations. Although family, friend, and teacher support may all contribute to the satisfaction of these needs, their relative relevance to particular needs may differ. Teacher support may be especially relevant to competence-need satisfaction because teachers regularly provide feedback, recognize students’ progress, and structure opportunities for academic achievement (Niemiec and Ryan, 2009). As materialism may serve as an alternative means of demonstrating achievement and personal worth, competence-need satisfaction fostered through teacher support may reduce adolescents’ reliance on material possessions as indicators of success. Empirical studies have consistently demonstrated a negative correlation between social support and materialism, suggesting that psychological and emotional support from close social ties is associated with less reliance on material possessions (Chaplin and John, 2010; Chia, 2010).

Despite this evidence, two critical gaps warrant further exploration. First, existing literature frequently portrays materialism as a uniform construct, overlooking the complexity and interconnectedness of its various dimensions. This reductionist perspective fails to capture the patterns of association among different components of materialistic orientations. Second, existing studies often examine a single source of support or aggregate support from parents, teachers, and peers into a composite measure (Estell and Perdue, 2013; Kristensen et al., 2023). Such approaches may obscure the distinct roles of different support sources, including the potentially underestimated role of teacher support (Harandi et al., 2017; Rueger et al., 2016). Although peer influence is often considered particularly salient during adolescence (Brechwald and Prinstein, 2011), teachers may also serve as important agents of value socialization. For instance, a six-year longitudinal study found that teachers were more influential than parents and friends in the development of adolescents’ societal interest (Wanders et al., 2021). These gaps highlight the need to examine how different sources of social support are associated with the interconnected components of adolescents’ materialistic values.

To address the aforementioned gaps, this study employed network analysis as a methodological framework, providing a means of simultaneously quantifying unique associations among multiple variables (Epskamp et al., 2018). Unlike traditional cluster analysis, which groups similar items based on shared characteristics, or factor analysis, which identifies latent dimensions underlying observed variables, network analysis conceptualizes psychological constructs as interconnected systems. This perspective allows for a more nuanced examination of individual features and structural interconnections among components (i.e., nodes). Such fine-grained modeling facilitates the examination of node centrality and the identification of bridge nodes connecting different parts of the estimated network (Borsboom and Cramer, 2013). Therefore, by employing network analysis, this study aims to characterize the pattern of conditional associations between social support and materialism.

The current study is centered on two primary objectives. First, it aims to identify the central components of materialism among adolescents. Second, it seeks to compare the conditional associations of family, teacher, and friend support with materialistic values and identify which source shows the strongest negative association within the estimated network. By comparing the distinct association patterns of these support sources, this research seeks to provide descriptive evidence that may inform future longitudinal and intervention studies.

## 2 Methods

### 2.1 Recruitment and sample

Using convenience sampling, participants were recruited from one junior high school and one senior high school in Hebei Province, China. The two schools were selected based on accessibility, and students in the participating classes were invited to take part in the study. Before data collection, the study protocol was approved by the Ethics Committee of Hebei University. Written informed consent was obtained from parents or legal guardians, and written assent was obtained separately from the adolescent participants. Administrative permission was also obtained from the participating schools. Participants were informed that participation was voluntary, that their responses would be kept confidential, and that they could withdraw from the study at any time without penalty.

The data were collected through paper-based questionnaires distributed in classrooms under the supervision of teachers. Teachers supervised the general administration of the questionnaires but did not participate in students’ responses or have access to individual completed questionnaires. Students completed the questionnaires independently. During the first assessment (Time 1), 1,976 participants completed the Perceived Social Support Scale (PSSS). After 7 months (Time 2), 1,666 of these participants completed the Material Values Scale (MVS), resulting in an 84.31% retention rate. In data cleaning, 27 participants were excluded because of high rates of missing data (missing rates > 30%). Therefore, the final sample size was reduced to 1,639. In the final analytic sample, participants’ ages at T1 ranged from 11 to 17 years (*M* = 14.49, *SD* = 1.11). The sample consisted of 52.50% female participants, with 31.90% living in urban areas and 8.20% being only children. To assess potential selection associated with follow-up attrition and subsequent data-quality exclusions, participants included in the final analytic sample (*n* = 1,639) were compared with those not included in the final analysis (*n* = 337) on their T1 demographic characteristics. No significant difference was found in family residence, *χ*^2^_(1)_ = 0.08, *p* = 0.782. However, participants not included in the final analysis (*M* = 14.10, *SD* = 1.24) were significantly younger than those included in the analytic sample (*M* = 14.49, *SD* = 1.11, *p* < 0.001, Cohen’s *d* = 0.35). The proportion of participants not included in the final analysis was also higher among male participants (20.33%, *n* = 198) than among female participants (13.94%, *n* = 139), *χ*^2^_(1)_ = 14.18, *p* < 0.001, ϕ = 0.08.

### 2.2 Measures

#### 2.2.1 Materialism

Materialism was assessed using the revised 13-item Material Values Scale (MVS; Li and Guo, 2009). The scale was originally developed to assess three dimensions: defining success through financial wealth, centrality of possessions, and pursuit of happiness via material acquisition. Participants indicated their level of agreement with each item. Responses were recorded on a 5-point Likert scale ranging from 1 (“*Strongly Disagree*”) to 5 (“*Strongly Agree*”). After reverse-scoring the relevant items, the mean score across all items was calculated to represent each participant’s level of materialism, yielding a theoretical range of 1 to 5. In the present sample, the observed scores ranged from 1.08 to 4.92. Higher scores indicated stronger materialistic tendencies. The revised MVS has been applied in Chinese adolescent samples (Song and Chen, 2019). In the present sample, a confirmatory factor analysis (CFA) indicated that the originally proposed three-factor structure did not fit the data adequately (CFI = 0.76, TLI = 0.70, RMSEA = 0.13, and SRMR = 0.09). The scale demonstrated acceptable internal consistency (Cronbach’s α = 0.81). For the primary network analysis, the 13 MVS items were entered as separate nodes rather than modeled as indicators of latent factors or aggregated into subscale scores.

#### 2.2.2 Social support

Social support was measured using the 12-item Perceived Social Support Scale (PSSS; Zimet et al., 1990). The scale consists of three four-item subscales assessing family, friend, and significant other support. To adapt the scale to the school context, the four items of the original “significant other support” subscale were modified to refer specifically to teachers, while the substantive content of the items was retained as closely as possible. For example, “There is a significant other who is around when I am in need” was changed to “There is a teacher who is around when I am in need.” Participants rated each item on a 7-point Likert scale ranging from 1 (“*Strongly Disagree*”) to 7 (“*Strongly Agree*”). Mean scores were calculated separately for each subscale, with possible scores ranging from 1 to 7. Higher scores indicated greater perceived social support. The Chinese version of the PSSS has demonstrated good psychometric properties (Jiang, 1999). Given the adaptation of the teacher-support subscale, we further examined the factor structure of the adapted PSSS in the present sample using CFA. The hypothesized three-factor model showed acceptable fit to the data, *χ*^2^_(51)_ = 466.14, *p* < 0.001, CFI = 0.95, TLI = 0.93, RMSEA = 0.07, and SRMR = 0.04. Standardized factor loadings ranged from 0.57 to 0.81 for family support, 0.77 to 0.83 for friend support, and 0.78 to 0.83 for teacher support, supporting the three-factor structure of the adapted scale. In this study, the Cronbach’s α coefficients were 0.72 for family support, 0.84 for friend support, and 0.85 for teacher support.

### 2.3 Statistical analyses

Statistical analyses were conducted using SPSS 27.0. Pearson’s correlation coefficients were calculated to examine the associations among the main study variables. Given that the main study variables were assessed using self-report measures, Harman’s single-factor test was conducted to assess potential common method bias. To examine the conditional associations between social support and materialism, we estimated a Gaussian Graphical Model (GGM) using R 4.4.1 (qgraph Version 1.10.1; bootnet Version 1.9.1; mice Version 3.19.0; Matrix Version 1.7–5; MASS Version 7.3–61). Prior to network estimation, skewness and kurtosis were examined for all 25 nodes. Skewness values ranged from −1.04 to 0.60, and kurtosis values ranged from −0.99 to 0.64 (see Supplementary Table 1). None of the nodes exceeded Kline’s (2011) thresholds for severe non-normality (|skewness| > 3 or |kurtosis| > 10). During data preprocessing, multiple imputation was performed using the mice package to address missing data. Twenty imputed datasets were generated (*m* = 20), with 10 iterations per imputation. Ordered Likert items were imputed with proportional odds logistic regression (*polr*) and continuous covariates with predictive mean matching (*pmm*). The imputation model included all 25 network nodes together with the covariates age, gender, parental education, household income, and family residence.

To reduce potential confounding, the network estimates were adjusted for participants’ age, gender, parental education, household income, and family residence. Specifically, within each of the 20 imputed datasets, each network node was residualized with respect to these covariates. Because network estimation was subsequently based on the continuous residualized scores, Pearson correlation matrices were computed within each imputed dataset and then pooled across the 20 datasets using Fisher’s z transformation. The resulting pooled residualized correlation matrix was used as the input for the primary network estimation. This procedure was used to characterize the linear associations among nodes after adjustment for the specified covariates. The 12 social-support nodes and 13 materialism nodes were entered into a single static network, in which edges represented undirected partial correlations between nodes conditional on all other nodes. Model selection was performed using the *ggmModSelect* function in the *qgraph* package (Epskamp et al., 2018). Using the pooled residualized correlation matrix, the extended Bayesian Information Criterion (EBIC) was applied with the hyperparameter set to γ = 0.25. The procedure first estimated 100 graphical lasso models across a logarithmically spaced sequence of λ values ranging from λmax to 0.01λmax (nlambda = 100; lambda.min.ratio = 0.01). The candidate network structures were subsequently refitted without regularization and compared using the EBIC. Starting from the best-fitting preliminary structure, the stepwise procedure iteratively added or removed edges until the EBIC could no longer be improved. Prior to estimation, the pooled correlation matrix was checked for positive definiteness and, where necessary, adjusted to a symmetric positive-definite matrix using the *nearPD* function. The precision matrix corresponding to the final unregularized solution was subsequently examined and confirmed to be positive definite (minimum eigenvalue = 0.22).

Centrality was assessed to evaluate the relative structural importance of individual nodes within the network. Nodes differ in their patterns of connectivity, with high-centrality nodes showing stronger, more numerous, or more strategically positioned connections with other nodes, depending on the centrality index examined. In contrast, low-centrality nodes generally exhibit fewer or weaker connections. To identify the most central nodes, we examined three commonly used centrality indices. First, betweenness centrality quantifies the number of shortest paths between all pairs of nodes that pass through a given node, reflecting its structural role in connecting different parts of the network (Opsahl et al., 2010). Second, closeness centrality assesses how closely a node is connected to all other nodes and is calculated as the inverse of the average shortest path length from that node to all others. Nodes with higher closeness have shorter overall paths to the other nodes in the estimated network (Opsahl et al., 2010). Finally, given the presence of both positive and negative associations in the network, expected influence (EI) was selected as the primary metric of node centrality. Expected influence extends traditional strength centrality by accounting for the sign of connections, making it particularly suitable for psychological networks involving negative edges (Robinaugh et al., 2016). This index represents the sum of all edge weights connected to a node and reflects the node’s overall signed connectivity with its immediate neighbors.

To compare the three support sources on an equal footing, we computed the bridge expected influence (BEI) for each of the 12 social-support items. BEI is defined as the signed sum of the edge weights connecting a given node to all nodes outside its own community (Jones et al., 2021). We designated the 13 materialism items as the target community and the 12 social-support items as the source community. For each social-support item, its BEI was calculated as the sum of its partial correlations with all 13 materialism nodes, thereby reflecting the net negative or positive connectivity between that support item and the materialism network. Subsequently, we averaged the BEI values across the four items within each support source (i.e., family, friend, and teacher) to obtain a source-level summary of bridge connectivity.

To evaluate whether the estimated edge weights differed from one another, bootstrapped difference tests were conducted using the *bootnet* package in R (Epskamp et al., 2018). This procedure computes pairwise differences in edge weights and applies nonparametric bootstrapping to generate 95% confidence intervals (CIs) for these differences. If a CI includes zero, the difference between the corresponding edge weights is considered statistically nonsignificant, indicating that the respective edges are not statistically distinguishable in magnitude. In addition, the stability of the centrality indices was evaluated using the case-dropping subset bootstrap method. This approach involves recalculating the network across progressively smaller random subsets of the original sample and comparing centrality estimates from each subset with those derived from the full dataset. Correlation coefficients between subset-derived and full-sample centrality estimates serve as an index of stability, with higher correlations indicating greater stability of the metric. The correlation stability coefficient (CS) was calculated as the maximum proportion of cases that could be dropped while retaining, with 95% probability, a correlation of at least 0.70 with the original centrality estimates. Values above 0.25 were considered acceptable, and values above 0.50 were considered preferable (Epskamp et al., 2018). All bootstrapping procedures were performed with 1,000 iterations. Because the bootstrap procedures require participant-level observations for repeated resampling, whereas the Fisher-z-pooled correlation matrix does not retain participant-level information, all bootstrap analyses were conducted using the residualized case-level data from the first imputed dataset. Accordingly, the bootstrap results were treated as ancillary assessments of source-level BEI differences, edge-weight accuracy, and centrality stability and did not incorporate between-imputation variability.

To enhance reproducibility, the analysis code, random seeds, variable-coding scheme, imputation settings, and network-estimation parameters were provided in the Supplementary materials.

## 3 Results

Harman’s single-factor test was conducted to evaluate potential common method bias (Zhou and Long, 2004). The unrotated exploratory factor analysis yielded six factors with eigenvalues greater than 1, with the first factor accounting for 20.72% of the total variance, below the commonly used criterion of 40%. Thus, no dominant single factor emerged, providing no indication that the results were dominated by a single common-method factor.

Table 1 presented the descriptive statistics and bivariate correlations among the main study variables. Teacher support and family support were significantly negatively associated with materialism, whereas friend support was not significantly associated with materialism. All reported network results were based on the residualized variables after adjustment for age, gender, parental education, household income, and family residence. In the estimated network, 65 out of 300 possible edges (21.67%) were retained as nonzero. The strongest positive edges were observed between Fr6 and Fr7 (edge weight = 0.52), Fr9 and Fr12 (0.46), and Fa3 and Fa4 (0.43), whereas the strongest negative edge was observed between MVS9 and MVS10 (−0.13). The complete edge-weight matrix was provided in Supplementary Table 2, and the network structure was visualized in Figure 1.

**Table 1:** Descriptive statistics and correlations for main study variables.

Variable	Materialism	Family support	Teacher support	Friend support
Materialism	1			
Family support	−0.11^**^	1		
Teacher support	−0.30^**^	0.38^**^	1	
Friend support	−0.01	0.34^**^	0.25^**^	1
*Mean*	2.80	5.14	3.76	4.98
*SD*	0.58	1.12	1.28	1.16
Min–max	1.08–4.92	1–7	1–7	1–7

All variables were presented as item-mean scores. Materialism was measured on a 1–5 scale, and the three social support subscales were measured on a 1–7 scale. ^**^p < 0.01.

**Figure 1:**
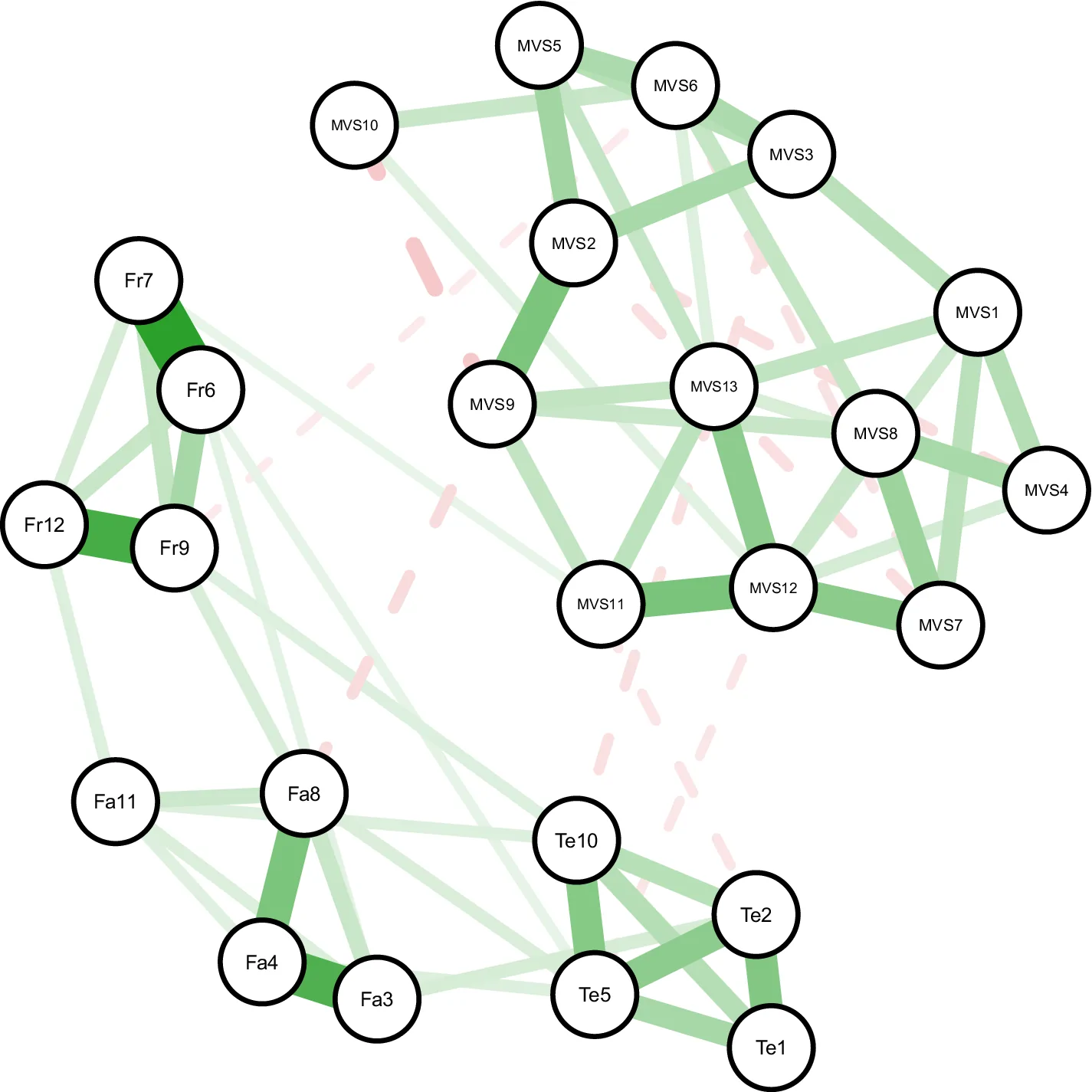
The network structure of perceived social support and materialism. Green edges represented positive associations between two nodes, whereas red dashed edges represented negative associations. MVS1 = “I admire people who own expensive homes, cars and clothes.” MVS2 = “I usually buy only the things I need.” MVS3 = “I put less emphasis on material things than most people I know.” MVS4 = “Some of the most important achievements in life include acquiring material possessions.” MVS5 = “I try to keep my life simple as far as possessions are concerned.” MVS6 = “I don’t place much emphasis on the amount of material objects people own as a sign of success.” MVS7 = “My life would be better if I owned certain things I don’t have.” MVS8 = “The things I own say a lot about how well I’m doing in life.” MVS9 = “I enjoy spending money on things that aren’t practical.” MVS10 = “I wouldn’t be any happier if I owned nicer things.” MVS11 = “Buying things gives me a lot of pleasure.” MVS12 = “I’d be happier if I could afford to buy more things.” MVS13 = “I like a lot of luxury in my life.” Te1 = “There is a teacher who is around when I am in need.” Te2 = “There is a teacher with whom I can share my joys and sorrows.” Fa3 = “My family really tries to help me.” Fa4 = “I get the emotional help and support I need from my family.” Te5 = “I have a teacher who is a real source of comfort to me.” Fr6 = “My friends really try to help me.” Fr7 = “I can count on my friends when things go wrong.” Fa8 = “I can talk about my problems with my family.” Fr9 = “I have friends with whom I can share my joys and sorrows.” Te10 = “There is a teacher in my life who cares about my feelings.” Fa11 = “My family is willing to help me make decisions.” Fr12 = “I can talk about my problems with my friends”.

As shown in Figure 1, the social support nodes were visually clustered largely apart from the materialism-related nodes, with most of the retained edges between the two groups representing negative partial correlations. The expected influence values were displayed in Figure 2. Because the centrality values were standardized, social-support items with values above or below zero represented nodes with expected influence above or below the network mean, respectively. The direction and magnitude of cross-community connectivity between social support and materialism were examined separately using bridge expected influence.

**Figure 2:**
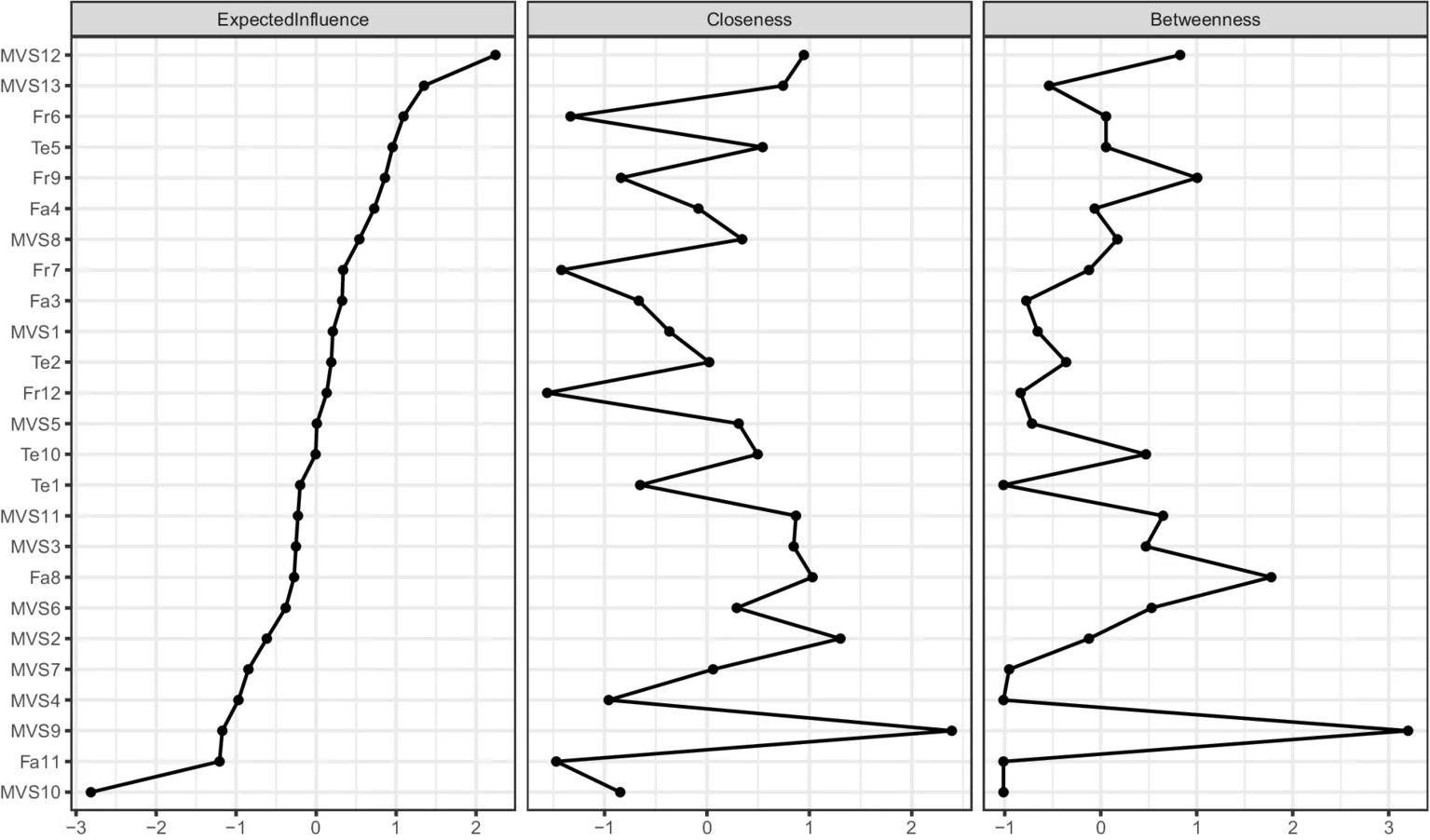
Standardized centrality measures. Values for centrality were standardized and sorted from least to most expected influence.

Among the materialism items, MVS12 (“I’d be happier if I could afford to buy more things”) exhibited the highest expected influence (standardized EI = 2.24), highlighting its central structural position in the network. To compare the three social-support sources, BEI was first calculated for each of the 12 support items with respect to the 13 materialism items. Mean BEI was most negative for teacher support (*M* = −0.05), followed by family support (*M* = −0.02) and friend support (*M* = 0.00). These results indicated that, within the 25-node network, teacher-support items collectively showed the strongest net negative connectivity with the materialism community.

Bootstrapped analyses were used to evaluate the accuracy of the edge-weight estimates and the stability of the centrality indices. Figure 3 presented the 95% bootstrapped confidence intervals for the edge weights, indicating that estimation precision varied across edges. Stability was further assessed using the case-dropping bootstrap procedure (Figure 4). The results indicated strong stability for expected influence (CS = 0.75), whereas closeness (CS = 0.28) and betweenness (CS = 0.20) showed limited stability. In addition, the bootstrapped edge-weight difference test (Figure 5) examined whether individual edge weights differed significantly from one another. Approximately 54.90% of pairwise comparisons of edge weights yielded confidence intervals that did not include zero, indicating statistically significant differences in edge-weight magnitude. These findings supported the interpretation of the estimated edge structure and expected influence, whereas closeness and betweenness should be interpreted cautiously.

**Figure 3:**
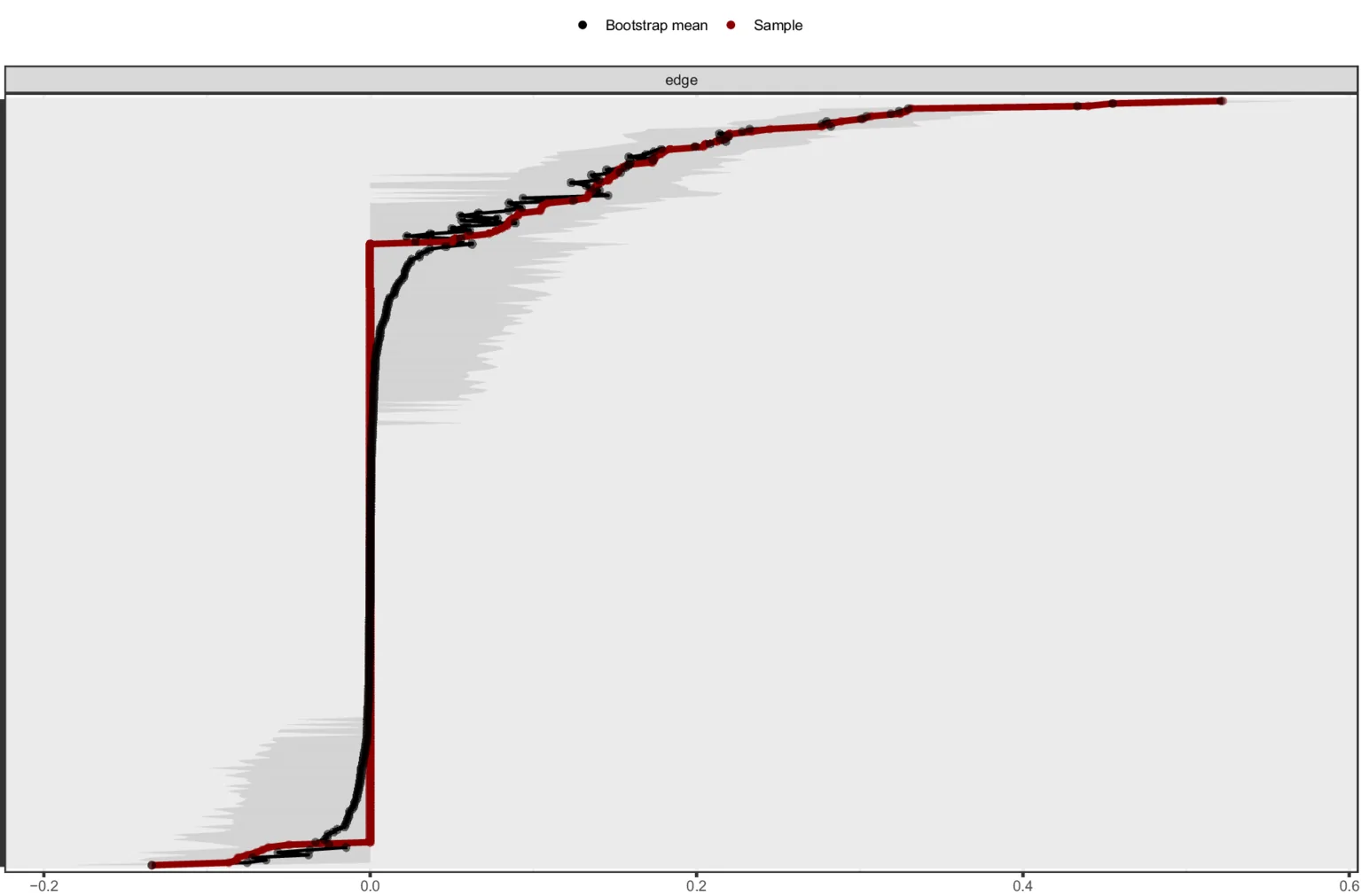
Bootstrapped confidence intervals of the edge weights. The red line indicated the estimated edge in the sample. The 95% bootstrap confidence intervals were shown in gray. The *x*-axis represented the edge weight values, and individual edges were shown as horizontal gray lines along the *y*-axis.

**Figure 4:**
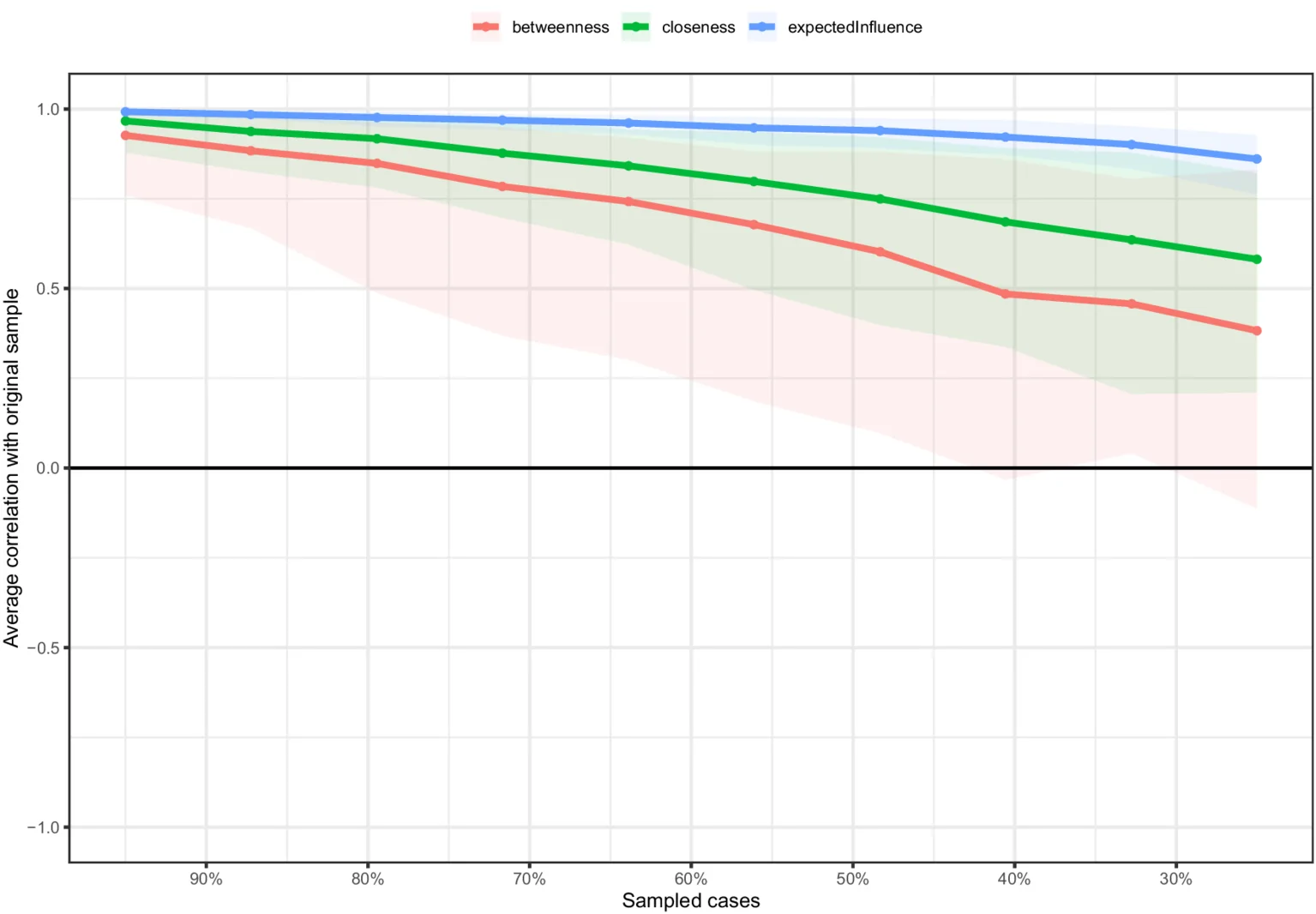
Network stability.

**Figure 5:**
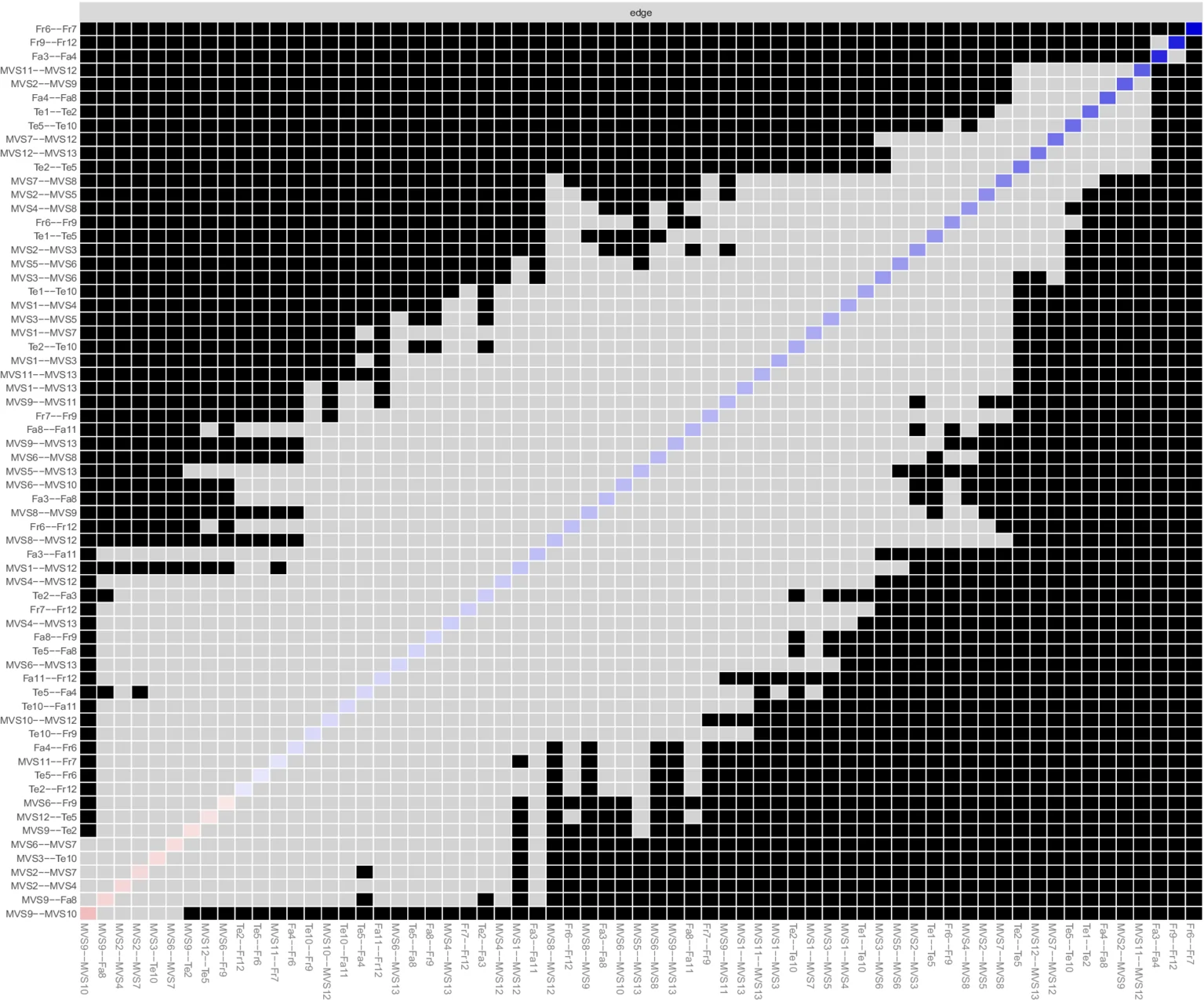
Bootstrapped edge-weight difference test. Black boxes at the intersections of rows and columns indicated that the bootstrapped 95% confidence interval of the difference between two edge weights excluded zero, whereas gray boxes indicated that it included zero.

Two sensitivity analyses were conducted to evaluate the robustness of the primary findings, using the same network-selection procedure as the primary analysis (*ggmModSelect*; EBIC γ = 0.25). In the materialism-only network comprising the 13 T2 materialism items, MVS12 and MVS13 again exhibited the two highest expected influence values (standardized EI = 2.11 and 1.17, respectively), indicating that their relatively central positions remained evident after the T1 social-support nodes were excluded. A complete-case ordinal network was also estimated using polychoric correlations among the 1,540 participants (93.96% of the analytic sample) with complete data on all 25 network items. MVS12 remained the materialism node with the highest expected influence (standardized EI = 2.68), and teacher support continued to show the most negative mean bridge expected influence with the materialism community (*M* = −0.07). These results supported the robustness of the principal findings across alternative network compositions and analytical specifications.

## 4 Discussion

This study utilized a network analysis approach to examine the central components of materialism and the associations of various sources of social support (i.e., family, teacher, and friend support) with adolescent materialistic tendencies. By examining node centrality within a network comprising both materialism-related items and social support items, the analysis identified central manifestations of materialism among adolescents, particularly those reflecting the desire to acquire more material possessions. Using item-level BEI values aggregated within each support source to compare the three sources, teacher-support items collectively showed the most negative mean BEI with the materialism community. This pattern reflects a comparatively stronger negative conditional association between teacher support and the materialism community. The study contributes to a nuanced understanding of the network relationships between social support and materialism by showing distinct patterns of cross-community connectivity across different sources of social support.

The case-dropping bootstrap analysis indicated strong stability for expected influence, whereas closeness and betweenness showed limited stability. Accordingly, substantive interpretations of node centrality focused primarily on expected influence, which reflects the overall magnitude and sign of a node’s connections with its immediate neighbors (Robinaugh et al., 2016). However, because the present network is static and undirected, these centrality estimates should be interpreted as descriptive structural characteristics rather than as indicators of causal influence. Given the limited stability of closeness and betweenness, interpretations based on these indices were treated as exploratory and should be considered cautiously.

More specifically, this study highlights several central manifestations of materialism among adolescents, particularly through items such as MVS12 (“I’d be happier if I could afford to buy more things”) and MVS13 (“I like a lot of luxury in my life”). These findings suggest that desires for acquiring more possessions and for luxury occupy relatively central positions among the materialism nodes within the estimated network. The desire for numerous and luxurious possessions may be associated with adolescents’ underlying psychological needs, such as the search for self-identity, social recognition, and a sense of belonging. During this critical stage of self-concept formation, materialistic tendencies may reflect adolescents’ efforts to construct and project an image that aligns with societal expectations or peer norms (Arnett, 1999; Luthar and Becker, 2002). Furthermore, the emphasis on luxury may reflect adolescents’ susceptibility to the pressures of consumer culture, which often associates luxury with success and status. This pattern may be associated with unrealistic expectations and a cycle of desire in which satisfaction is fleeting and contingent upon the acquisition of increasingly high-status items. As identity development, social-recognition motives, peer norms, and exposure to consumer culture were not directly examined, these interpretations remain tentative hypotheses rather than mechanisms established by the present findings. While previous research has explored materialistic values among adolescents (Chaplin and John, 2007; Goldberg et al., 2003), it has often paid insufficient attention to the relative prominence of specific manifestations of materialism during adolescence. By focusing on this developmental stage, the current study addresses this gap, highlighting the relatively central positions of desires for greater and more luxurious possessions in the estimated network, thereby providing a more nuanced understanding of materialism in this age group.

In contrast to MVS12 and MVS13, several other materialism nodes, including MVS10 (“I wouldn’t be any happier if I owned nicer things”), MVS9 (“I enjoy spending money on things that aren’t practical”), MVS7 (“My life would be better if I owned certain things I don’t have”), MVS4 (“Some of the most important achievements in life include acquiring material possessions”), and MVS2 (“I usually buy only the things I need”), showed relatively low EI. These items encompass heterogeneous aspects of materialism, including the perceived link between possessions and happiness, the practical utility of consumption, and the association of material possessions with achievement. Their lower EI suggests that these aspects occupy less central positions within the estimated network, consistent with network perspectives that distinguish relatively central from more peripheral components of psychological constructs (Bringmann and Eronen, 2018; Robinaugh et al., 2016). Thus, different materialistic beliefs and preferences appear to occupy different structural positions rather than contributing equally to the estimated network.

Additionally, MVS9 (“I enjoy spending money on things that aren’t practical”) showed a relatively low expected influence. Although MVS9 had relatively higher closeness and betweenness values, both indices showed limited stability in the case-dropping bootstrap analyses. Accordingly, we do not draw substantive conclusions about MVS9 as a structural connector. This pattern is reported only as a descriptive and exploratory observation and should be interpreted cautiously pending replication.

Through a detailed network analysis of different sources of social support, this study found that teacher-support items showed the strongest net negative bridge connectivity with materialism, as indexed by mean BEI. This pattern indicates a stronger negative conditional association between teacher support and adolescents’ materialistic values. Against this background, the stronger teacher-support association may be considered in relation to the role of teacher-student interactions in adolescents’ value development (Eccles and Roeser, 2011; Steinberg and Morris, 2001). Teachers occupy a formal and structured position within the school environment and establish classroom expectations and norms, provide feedback, recognize students’ progress, and emphasize academic achievement, personal growth, and social contribution. These interactions may provide contexts in which adolescents encounter perspectives that place less emphasis on material possessions and greater emphasis on intrinsic goals. Teacher-student interactions may also be associated with adolescents’ critical reflection and their evaluation of the importance of material possessions (Blazar and Kraft, 2017; Wentzel, 2012). From the perspective of self-determination theory (Ryan and Deci, 2000), teacher support may be related to adolescents’ experiences of competence through feedback and opportunities for achievement. Such experiences may be associated with the extent to which material possessions are viewed as indicators of achievement or self-worth (Niemiec and Ryan, 2009). In contrast, family and friend support may be more closely related to needs for relatedness and emotional security, which may help contextualize the relatively similar mean BEI values observed for these two sources. Taken together, these perspectives provide plausible interpretations of the observed network pattern, but the corresponding processes remain untested and should not be construed as mechanisms established by the present findings. Overall, these findings highlight the potentially distinctive association between teacher support and adolescent materialistic values. They also underscore the importance of examining different sources of social support separately rather than combining them into a single composite measure.

Despite the valuable insights generated by our study, it is subject to several limitations that merit consideration when interpreting the findings. First, both materialistic values and perceived social support were assessed using self-report questionnaires administered in classroom settings, making the findings susceptible to social desirability bias and common method variance. Although the constructs were assessed at different waves and Harman’s single-factor test did not indicate a dominant common-method factor, common method variance cannot be completely ruled out. Participants’ tendency to respond in accordance with perceived social norms, together with the shared response format and administration context, may have inflated or otherwise distorted the observed associations. Future studies should incorporate multiple information sources or alternative measurement methods to reduce these potential biases. Second, this study used a convenience sample from one junior high school and one senior high school in Hebei Province, which limits the generalizability of the findings to adolescents from other regions and school contexts. Selective attrition was also observed for age and gender, with younger adolescents and boys being more likely to be excluded from the final analytic sample. Although age and gender were included as covariates in the primary network analysis, this adjustment cannot fully eliminate potential selection bias resulting from differential attrition. In addition, students were nested within classes and schools and shared teachers, peer environments, and institutional norms, whereas the present network analysis treated observations as independent. This unmodeled dependency may have affected the estimated associations and their precision. Because only two schools were included, school-level clustering could not be reliably estimated or modeled. Future studies should recruit participants from a broader range of schools and classes and employ multilevel or other cluster-aware network methods. Third, although social support was assessed at T1 and materialism at T2, materialism was not assessed at T1, and social support was not reassessed at T2. The T1 social-support items and T2 materialism items were entered into a single static network based on undirected partial correlations. Consequently, the observed associations do not establish temporal, directional, or causal relationships. Future longitudinal studies assessing both social support and materialism at multiple time points are needed to clarify their temporal and potentially directional relationships. Finally, the seven-month interval was not selected on the basis of a specific theoretical rationale and represented only a single fixed lag. Because the study included only two waves and each construct was assessed at only one wave, sensitivity analyses comparing shorter and longer intervals could not be conducted. It therefore remains unclear whether a similar network structure would emerge under alternative assessment intervals. Future multiwave studies should repeatedly assess both constructs across different time intervals to examine the temporal robustness of the network structure.

## 5 Conclusion

This study utilized network analysis to examine the structural characteristics of adolescent materialism and its associations with social support. The analysis identified MVS12 and MVS13 as relatively central materialism nodes within the estimated network, highlighting the prominence of desires for acquiring more possessions and for luxury. Additionally, in the item-level network, teacher-support items showed the most negative mean BEI with the materialism community, compared with family-support items and friend-support items. These findings highlight teacher support as a potentially important correlate of adolescent materialism and underscore the relevance of teacher support to understanding adolescents’ materialistic values.

## Data Availability

The raw data supporting the conclusions of this article will be made available by the authors, without undue reservation.
