# Supplementary material for: Associations between social support and central components of adolescent materialism: a network analysis
Source: Front Psychol. 2026 Oct 14;17:1925963. doi: 10.3389/fpsyg.2026.1925963 (PMC13713670; doi:10.3389/fpsyg.2026.1925963)
Supplement: Supplementary file 1 [file Data_Sheet_1.PDF]

25-Node Network Analysis (Materialism + Social Support)

1. Overview

This document contains the public release of the R script used for a supplementary network analysis of adolescent materialism and perceived social support. The analysis estimates a 25-node Gaussian graphical model (GGM) in which 13 materialism items and 12 perceived social support items are entered as separate nodes, and it reports edge weights, node centrality (expected influence, closeness, betweenness) and bridge expected influence (BEI) between the social support items and the materialism items.

All estimates are adjusted for sociodemographic covariates by residualizing every node on the average of two parental-education indicators, monthly household income, gender, age and family residence before the network is estimated. Multiple imputation is used to handle item-level missing data, and the resulting correlation matrices are pooled with Fisher z averaging.

2. Analysis pipeline

- 1. Read the SPSS data file and select the 25 item variables plus the six covariates.
- 2. Clean missing values: social support items are scored 1-7 and materialism items 1-5; out-of-range codes and the code 9 are set to NA.
- 3. Multiple imputation with mice (m = 20 imputations, 10 iterations, proportional odds logistic regression for ordinal items and predictive mean matching for numeric items).
- 4. Residualize each of the 25 nodes on all covariates, separately for every imputed dataset.
- 5. Compute a correlation matrix for each imputed dataset with qgraph::cor_auto.
- 6. Pool the correlation matrices with Fisher z averaging and, if necessary, correct the pooled matrix to the nearest positive-definite matrix.
- 7. Estimate the network with qgraph::ggmModSelect using the EBIC with gamma = 0.25.
- 8. Diagnose the precision matrix of the final unregularized solution, including its minimum eigenvalue.
- 9. Visualize the network and compute node-level centrality indices.
- 10. Compute bridge expected influence for each of the 12 support items with respect to the 13 materialism items.
- 11. Bootstrap the mean BEI of each support source and test pairwise differences.
- 12. Nonparametric bootstrap for edge-weight confidence intervals and case-dropping bootstrap for centrality stability.
- 13. Export centrality values and figures.

3. Software requirements

The script was developed and run under R 4.4.1 (x86_64-apple-darwin20). The following packages are required; the script installs any that are missing. Version numbers are those used for the reported results.

Package	Version	Package	Version
haven	2.5.5	dplyr	1.2.1
stringr	1.5.1	mice	3.19.0
qgraph	1.10.1	bootnet	1.9.1
Matrix	1.7-5	matrixcalc	1.0-6
psych	2.6.5	ggplot2	4.0.3
readr	2.1.5	tidyr	1.3.2
foreach	1.5.2	doParallel	1.0.17
networktools	1.6.0	glasso	1.11

4. Inputs and outputs

Input

- data.sav - an SPSS file containing the item responses and the covariates.

Outputs (written to the directory given by out_dir)

- The estimated edge-weight matrix and a ranked edge list.
- Centrality values in raw and standardized form, and the data behind the centrality figure.
- Item-level and source-level bridge expected influence, plus the bootstrap difference test.
- The precision-matrix diagnostics of the final unregularized solution.
- Edge-weight confidence intervals, centrality stability coefficients and all figures.
- The imputation object and the fitted network objects, so that later steps can be re-run without repeating the imputation.

5. Running the script

Edit the two path constants at the beginning of section 0 so that out_dir points to the directory in which results should be written and data_path points to the SPSS file, then run:

```
Rscript 25node_network_analysis_public.R
```

The number of bootstrap iterations and the number of worker cores can be overridden through environment variables. The defaults are 1000 iterations and 4 cores.

```
NBOOTS=1000 NCORES=4 Rscript 25node_network_analysis_public.R
```

Runtime note: the network-selection step is computationally heavy and its cost varies considerably across bootstrap resamples, so a full run with 1000 iterations may take several hours. Set NBOOTS to a small value (for example 10) for a quick smoke test. Because the imputation object is cached in the output directory, re-running the script after the first pass does not repeat the imputation.

6. Script listing

Section headings below correspond to the numbered sections of the script.

```
##############################################################################
## Supplementary network analysis: 25-node network
##   13 materialism items + 12 perceived social support items
##
## Pipeline:
##   multiple imputation -> node-wise residualization -> correlation pooling
##   -> Gaussian graphical model selection -> centrality and bridge centrality
##   -> bootstrap accuracy and stability checks
##
## Before running, edit the two path constants in section 0.
##############################################################################
```

0. Setup

```
# Output directory (created automatically if it does not exist)
out_dir <- "output"
if (!dir.exists(out_dir)) dir.create(out_dir, recursive = TRUE)

# Input data file (SPSS .sav)
data_path <- "data.sav"

## ---- Run-size configuration ----
## Affects only the number of bootstrap iterations and the number of worker
## cores; it does not change any statistical setting or random seed.
## Defaults: 1000 iterations and 4 cores. Override through environment
## variables for a quick smoke test, e.g.:
N_BOOTS <- as.integer(Sys.getenv("NBOOTS", unset = "1000"))
N_CORES <- as.integer(Sys.getenv("NCORES", unset = "4"))
cat("Run configuration: N_BOOTS =", N_BOOTS, "| N_CORES =", N_CORES, "\n")

set.seed(20260805)  # Fixed seed for reproducibility

# Load required packages (installed automatically if missing)
pkgs <- c("haven", "dplyr", "stringr", "mice", "qgraph", "bootnet",
          "Matrix", "matrixcalc", "psych", "ggplot2", "readr", "tidyr",
          "foreach", "doParallel", "networktools")
to_install <- pkgs[!sapply(pkgs, requireNamespace, quietly = TRUE)]
if (length(to_install) > 0) install.packages(to_install, dependencies = TRUE)

if (!requireNamespace("glasso", quietly=TRUE)) install.packages("glasso")
library(haven)
library(dplyr)
library(stringr)
library(mice)
library(qgraph)
library(bootnet)
library(Matrix)
library(matrixcalc)
library(psych)
library(ggplot2)
library(readr)
library(tidyr)
library(foreach)
library(doParallel)
library(networktools)
library(glasso)

# Save sessionInfo
sink(file.path(out_dir, "sessionInfo.txt"))
cat("=== sessionInfo() ===\n")
print(sessionInfo())
sink()
```

1. Read data and define variables

```
raw <- read_sav(data_path) %>% haven::zap_labels()

# Raw item variable names
```

```
teacher_vars <- c("D01T1", "D02T1", "D05T1", "D10T1")
family_vars  <- c("D03T1", "D04T1", "D08T1", "D11T1")
friend_vars  <- c("D06T1", "D07T1", "D09T1", "D12T1")
psss_vars    <- c(teacher_vars, family_vars, friend_vars)
mvs_vars     <- sprintf("F%02d", 1:13)   # F01~F13
all_item_vars <- c(psss_vars, mvs_vars)

# Socioeconomic status and additional control variables
ses_vars <- c("A07T1", "A08T1", "A09T1")  # paternal education, maternal education, household income
extra_controls <- c("A01T1", "A03T1", "A04T1")  # gender, age, family residence

# Keep only the variables required for the analysis
dat0 <- raw %>% dplyr::select(dplyr::all_of(c(all_item_vars, ses_vars, extra_controls)))

```

2. Missing-value cleaning

```
# Social support: code 9 and values outside 1-7 are treated as missing
dat0 <- dat0 %>%
  mutate(across(all_of(psss_vars), ~as.numeric(.x))) %>%
  mutate(across(all_of(psss_vars), ~ifelse(.x == 9, NA, .x))) %>%
  mutate(across(all_of(psss_vars), ~ifelse(.x < 1 | .x > 7, NA, .x)))

# Materialism: values outside 1-5 are treated as missing
dat0 <- dat0 %>%
  mutate(across(all_of(mvs_vars), ~as.numeric(.x))) %>%
  mutate(across(all_of(mvs_vars), ~ifelse(.x < 1 | .x > 5, NA, .x)))

# Cleaning of the socioeconomic status variables
dat0 <- dat0 %>%
  mutate(
    A07T1 = as.numeric(A07T1),
    A08T1 = as.numeric(A08T1),
    A09T1 = as.numeric(A09T1)
  ) %>%
  mutate(
    A07T1 = ifelse(A07T1 == 9, NA, A07T1),  # 9 = missing
    A08T1 = ifelse(A08T1 == 9, NA, A08T1)
    # A09T1 is already NA when blank
  )

# Cleaning of the additional control variables
dat0 <- dat0 %>%
  mutate(
    A01T1 = as.numeric(A01T1),
    A03T1 = as.numeric(A03T1),
    A04T1 = as.numeric(A04T1)
  ) %>%
  mutate(
    A01T1 = ifelse(A01T1 == 9, NA, A01T1),   # 9 = missing
    A04T1 = ifelse(A04T1 == 9, NA, A04T1)    # 9 = missing
    # A03T1 is already NA when blank
  )

# Missing-rate overview
miss_summary <- sapply(dat0, function(x) mean(is.na(x)))
miss_df <- data.frame(var = names(miss_summary), missing_rate = as.numeric(miss_summary)) %>%
  arrange(desc(missing_rate))
readr::write_csv(miss_df, file.path(out_dir, "missing_rate_by_variable.csv"))
print(miss_df)

```

3. Multiple imputation (socioeconomic status included)

```
to_ord <- function(x, levels_vec) {
  ordered(x, levels = levels_vec)
}

dat_imp <- dat0
for (v in psss_vars) dat_imp[[v]] <- to_ord(dat_imp[[v]], 1:7)
for (v in mvs_vars)  dat_imp[[v]] <- to_ord(dat_imp[[v]], 1:5)
dat_imp$A07T1 <- to_ord(dat_imp$A07T1, 1:6)
dat_imp$A08T1 <- to_ord(dat_imp$A08T1, 1:6)
dat_imp$A01T1 <- to_ord(dat_imp$A01T1, 1:2)   # gender: 1/2
```

```r
dat_imp$A04T1 <- to_ord(dat_imp$A04T1, 1:2)   # family residence: 1/2
# A09T1 and A03T1 remain numeric

# mice settings
ini <- mice(dat_imp, m = 1, maxit = 0, printFlag = FALSE)
meth <- ini$method
pred <- ini$predictorMatrix

meth[psss_vars] <- "polr"
meth[mvs_vars]  <- "polr"
meth["A07T1"]   <- "polr"
meth["A08T1"]   <- "polr"
meth["A09T1"]   <- "pmm"
meth["A01T1"]   <- "polr"   # gender
meth["A04T1"]   <- "polr"   # family residence
meth["A03T1"]   <- "pmm"    # age

pred[,] <- 1
diag(pred) <- 0

# Run the imputation (adjust m and maxit to the available hardware)
imp_file <- file.path(out_dir, "imp_25node_m20.rds")

if (file.exists(imp_file)) {
  cat("Loading cached imputation object:", imp_file, "\n")
  imp <- readRDS(imp_file)
} else {
  cat("No cached imputation object found; running multiple imputation...\n")
  imp <- mice(dat_imp, m = 20, maxit = 10,
              method = meth, predictorMatrix = pred,
              seed = 20260805, printFlag = TRUE)
  saveRDS(imp, imp_file)
  cat("Imputation completed and saved to:", imp_file, "\n")
}

# Imputation diagnostics plot
pdf(file.path(out_dir, "MI_diagnostics_traceplot.pdf"), width = 12, height = 8)
plot(imp)
dev.off()

```

4. Residualization of every node

```r
residualize_nodes <- function(df_completed) {
  # Coerce to numeric
  to_num <- function(x) {
    if (is.ordered(x) || is.factor(x)) {
      as.numeric(as.character(x))
    } else {
      as.numeric(x)
    }
  }
  num_df <- as.data.frame(lapply(df_completed, to_num))

  # Average of the two parental-education indicators
  parent_edu <- rowMeans(num_df[, c("A07T1", "A08T1")], na.rm = TRUE)
  income     <- num_df$A09T1
  gender     <- num_df$A01T1          # 1 = male, 2 = female
  age        <- num_df$A03T1
  location   <- num_df$A04T1          # 1 = urban, 2 = rural

  # Build the 25 nodes
  mvs_df <- num_df[, mvs_vars, drop = FALSE]
  colnames(mvs_df) <- paste0("MVS", 1:13)

  support_df <- num_df[, psss_vars, drop = FALSE]
  colnames(support_df) <- c(
    "Te1", "Te2", "Te5", "Te10",    # teacher
    "Fa3", "Fa4", "Fa8", "Fa11",    # family
    "Fr6", "Fr7", "Fr9", "Fr12"     # friend
  )

  node_df <- cbind(mvs_df, support_df)

  # Residualize every node on all covariates
```

```r
  residual_list <- lapply(node_df, function(y) {
    dat_temp <- data.frame(
      y = y,
      parent_edu = parent_edu,
      income = income,
      gender = gender,
      age = age,
      location = location
    )
    complete_idx <- complete.cases(dat_temp)
    if (sum(complete_idx) < 50) {
      warning("Too few complete cases; residualization may be unstable")
    }
    model <- lm(y ~ parent_edu + income + gender + age + location,
                data = dat_temp, na.action = na.exclude)
    residuals(model)
  })

  residual_df <- as.data.frame(residual_list)
  colnames(residual_df) <- colnames(node_df)

  return(residual_df)
}

# Residualize every imputed dataset
completed_list <- mice::complete(imp, action = "all")
node_list_resid <- lapply(completed_list, residualize_nodes)

N <- nrow(node_list_resid[[1]])
cat("Sample size after residualization, N =", N, "\n")
stopifnot(all(sapply(node_list_resid, nrow) == N))

# Check for an unusual amount of NA after residualization
na_check <- sapply(node_list_resid[[1]], function(x) mean(is.na(x)))
cat("NA proportion per node after residualization (should be close to 0):\n")
print(round(na_check, 4))

```

5. Correlation matrix per imputed dataset (cor_auto)

```r
cor_list <- lapply(node_list_resid, function(df) {
  # Residuals are continuous, so cor_auto remains appropriate
  qgraph::cor_auto(df)
})

```

6. Fisher z-averaged pooling of the correlation matrices

```r
pool_cor_fisherz <- function(R_list) {
  m <- length(R_list)
  p <- ncol(R_list[[1]])
  Rz_sum <- matrix(0, p, p)
  for (i in 1:m) {
    R <- R_list[[i]]
    diag(R) <- 0
    Rz_sum <- Rz_sum + atanh(pmax(pmin(R, 0.999999), -0.999999))
  }
  Rz <- Rz_sum / m
  R_pool <- tanh(Rz)
  diag(R_pool) <- 1
  R_pool <- (R_pool + t(R_pool)) / 2
  colnames(R_pool) <- colnames(R_list[[1]])
  rownames(R_pool) <- rownames(R_list[[1]])
  return(R_pool)
}

R_pool <- pool_cor_fisherz(cor_list)

# Positive-definiteness check and correction
if (!matrixcalc::is.positive.definite(R_pool)) {
  cat("Correlation matrix is not positive definite; applying the nearPD correction...\n")
  R_pool <- as.matrix(Matrix::nearPD(R_pool, corr = TRUE)$mat)
}

```

```
readr::write_csv(as.data.frame(round(R_pool, 3)),
                 file.path(out_dir, "pooled_correlation_matrix_residualized.csv"))

```

7. Network estimation (ggmModSelect)

```
ggmModSelect_safe <- function(S, n, gamma = 0.25, verbose = TRUE,
                              nLambda = 100, lambda.min.ratio = 0.01,
                              stepup = TRUE, stepdown = TRUE) {
  fm <- names(formals(qgraph::ggmModSelect))
  args <- list()
  if ("S" %in% fm) {
    args$S <- S
  } else if ("corMat" %in% fm) {
    args$corMat <- S
  } else {
    stop("Neither S nor corMat is an argument of qgraph::ggmModSelect(); check the package version.")
  }
  if ("n" %in% fm) args$n <- n
  if ("gamma" %in% fm) args$gamma <- gamma
  if ("verbose" %in% fm) args$verbose <- verbose
  if ("nLambda" %in% fm) args$nLambda <- nLambda
  if ("lambda.min.ratio" %in% fm) args$lambda.min.ratio <- lambda.min.ratio
  if ("stepup" %in% fm) args$stepup <- stepup
  if ("stepdown" %in% fm) args$stepdown <- stepdown
  fit <- do.call(qgraph::ggmModSelect, args)
  return(fit)
}

get_W <- function(fit) {
  if (inherits(fit, "qgraph")) return(qgraph::getWmat(fit))
  if (!is.null(fit$graph)) return(fit$graph)
  if (!is.null(fit$results$graph)) return(fit$results$graph)
  stop("Could not extract the edge-weight matrix from the fitted object; check its structure.")
}

fit_modselect <- ggmModSelect_safe(S = R_pool, n = N, gamma = 0.25,
                                   nLambda = 100, lambda.min.ratio = 0.01,
                                   stepup = TRUE, stepdown = TRUE)

W <- get_W(fit_modselect)
colnames(W) <- rownames(W) <- colnames(R_pool)

# Save the edge-weight matrix
W_out <- round(W, 3)
readr::write_csv(as.data.frame(W_out),
                 file.path(out_dir, "edge_weight_matrix_full_residualized.csv"))

# Edge list sorted by absolute weight
edge_table <- function(Wmat) {
  labs <- colnames(Wmat)
  idx <- which(upper.tri(Wmat) & abs(Wmat) > 0, arr.ind = TRUE)
  data.frame(node1 = labs[idx[,1]], node2 = labs[idx[,2]],
             partial_r = Wmat[idx]) %>%
    arrange(desc(abs(partial_r)))
}
edges_rank <- edge_table(W) %>% mutate(partial_r = round(partial_r, 3))
readr::write_csv(edges_rank, file.path(out_dir, "edge_weight_ranked_residualized.csv"))
readr::write_csv(head(edges_rank, 20), file.path(out_dir, "top20_edges_residualized.csv"))


################################################################################
```

7.5 Precision-matrix diagnostics

```
##
## The precision matrix of the final unregularized solution is obtained by
## refitting the selected structure without regularization (rho = 0). It is
## NOT the same object as solve(R_pool) (the saturated solution) nor as the
## partial-correlation weight matrix, which is not a precision matrix at all
## because its diagonal is zero. All three are reported below with explicit
## labels so that they cannot be confused.
##
## The zero pattern passed to glasso must exclude the diagonal: glasso assigns
```

```
## a very large penalty to every position listed in 'zero' and penalizes the
## diagonal by default (penalize.diagonal = TRUE), which would drive the
## diagonal of the precision matrix to approximately zero.
###############################################################################

cat("\n========== 7.5 Precision-matrix diagnostics ==========\n")

# 1. Symmetrize the edge-weight matrix and set its diagonal to zero
W_sym <- (W + t(W)) / 2
diag(W_sym) <- 0
p <- ncol(W_sym)
node_ids <- colnames(W_sym)

# 2. Selected structure (the diagonal must never enter the zero pattern)
selected_structure <- abs(W_sym) > 1e-8
diag(selected_structure) <- FALSE

# 3. Zero pattern: unselected off-diagonal positions only
zero_idx <- which(selected_structure == FALSE &
                    row(selected_structure) != col(selected_structure),
                  arr.ind = TRUE)
stopifnot(nrow(zero_idx) == 0 || all(zero_idx[, 1] != zero_idx[, 2]))  # no diagonal entry in the zero pattern

n_edges <- sum(selected_structure[upper.tri(selected_structure)])
cat("Number of nodes, p =", p, "\n")
cat("Retained (nonzero) edges =", n_edges, "/", p * (p - 1) / 2,
    "(", round(n_edges / (p * (p - 1) / 2) * 100, 2), "% )\n")
cat("Zero-pattern positions (off-diagonal only) =", nrow(zero_idx),
    "| of which on the diagonal =", sum(zero_idx[, 1] == zero_idx[, 2]), "\n")

# 4. Unregularized refit of the selected structure -> final precision matrix
glasso_fit <- glasso::glasso(s = R_pool,
                             rho = 0,                  # 0 = no regularization
                             zero = zero_idx,          # off-diagonal zero constraints only
                             penalize.diagonal = FALSE)  # leave the diagonal untouched
Theta <- (glasso_fit$wi + t(glasso_fit$wi)) / 2         # numerical symmetrization
dimnames(Theta) <- list(node_ids, node_ids)

# 5. Shared eigenvalue and positive-definiteness helpers
sym <- function(M) (M + t(M)) / 2
min_eig <- function(M) {
  M <- sym(M)
  min(eigen(M, symmetric = TRUE, only.values = TRUE)$values)
}
safe_pd <- function(M) {
  M <- sym(M)
  isTRUE(tryCatch(matrixcalc::is.positive.definite(M), error = function(e) FALSE))
}

Sinv <- sym(solve(R_pool))

diag_checks <- data.frame(
  matrix = c(
    "Theta = precision matrix of the FINAL unregularized solution (selected structure)",
    "solve(R_pool) = precision matrix of the saturated unregularized solution",
    "W_sym = partial-correlation weight matrix (diagonal = 0); NOT a precision matrix"
  ),
  is_precision_matrix = c(TRUE, TRUE, FALSE),
  min_eigenvalue      = round(c(min_eig(Theta), min_eig(Sinv), min_eig(W_sym)), 6),
  positive_definite   = c(safe_pd(Theta), safe_pd(Sinv), safe_pd(W_sym)),
  stringsAsFactors    = FALSE
)

cat("\n--- Minimum eigenvalue of each matrix, clearly labelled ---\n")
print(diag_checks, row.names = FALSE)

cat("\n>>> Minimum eigenvalue of the final unregularized precision matrix:\n")
cat("    Theta =",
    round(min_eig(Theta), 4), "\n")
cat("    Theta is positive definite =", safe_pd(Theta),
    "| max |Theta_ij| at constrained positions =", round(max(abs(Theta[zero_idx])), 12), "\n")
cat("    First five diagonal entries of Theta:",
    paste(signif(diag(Theta)[1:5], 4), collapse = ", "), "\n")

readr::write_csv(diag_checks,
```

```r
                  file.path(out_dir, "precision_matrix_diagnostics.csv"))
saveRDS(list(Theta = Theta, W_sym = W_sym, zero_idx = zero_idx),
        file.path(out_dir, "final_precision_matrix_theta.rds"))

# Proportion of retained (nonzero) edges
n_nonzero <- sum(abs(W[upper.tri(W)]) > 0)
n_possible <- p * (p - 1) / 2
pct <- round(n_nonzero / n_possible * 100, 2)
cat("\nNumber of nonzero edges:", n_nonzero, "\n")
cat("Proportion:", pct, "%\n")


```

8. Network visualization

```r
groups <- list(
  Materialism = colnames(W)[1:13],
  Teacher     = colnames(W)[14:17],
  Family      = colnames(W)[18:21],
  Friend      = colnames(W)[22:25]
)
group_colors <- c(Materialism = "#4C78A8", Teacher = "#E58606",
                  Family = "#54A24B", Friend = "#D62728")

set.seed(20260805)
layout0 <- qgraph::qgraph(W, layout = "spring", labels = colnames(W),
                          groups = groups, color = unname(group_colors),
                          vsize = 5, label.cex = 0.7, label.scale = FALSE,
                          edge.color = ifelse(W > 0, "#2CA02C", "#D62728"),
                          negDashed = TRUE, legend = TRUE, legend.cex = 0.6,
                          border.width = 1.2, cut = 0, minimum = 0,
                          details = TRUE)$layout

png(file.path(out_dir, "Figure_network_residualized.png"),
    width = 3200, height = 2400, res = 300)
qgraph::qgraph(W, layout = layout0, labels = colnames(W),
               groups = groups, color = unname(group_colors),
               vsize = 5, label.cex = 0.7,
               edge.color = ifelse(W > 0, "#2CA02C", "#D62728"),
               negDashed = TRUE, border.width = 1.2, cut = 0, minimum = 0,
               legend = TRUE, legend.cex = 0.6,
               title = "Residualized Network")
dev.off()

pdf(file.path(out_dir, "Figure_network_residualized.pdf"), width = 14, height = 10)
qgraph::qgraph(W, layout = layout0, labels = colnames(W),
               groups = groups, color = unname(group_colors),
               vsize = 5, label.cex = 0.7,
               edge.color = ifelse(W > 0, "#2CA02C", "#D62728"),
               negDashed = TRUE, border.width = 1.2, cut = 0, minimum = 0,
               legend = TRUE, legend.cex = 0.6,
               title = "Residualized Network")
dev.off()

```

9. Centrality analysis (expected influence)

```r
cent <- qgraph::centrality(W)
saveRDS(cent, file.path(out_dir, "centrality_object_residualized.rds"))

# Expected influence computed manually (signed row sums)
EI <- rowSums(W)
ei_df <- data.frame(node = names(EI), expected_influence = as.numeric(EI)) %>%
  arrange(desc(expected_influence))
readr::write_csv(ei_df, file.path(out_dir, "expected_influence_rank_residualized.csv"))

mvs_ei <- ei_df %>% filter(grepl("^MVS", node)) %>% arrange(desc(expected_influence))
cat("\n=== Expected influence ranking of the materialism items ===\n")
print(head(mvs_ei, 5))
write.csv(mvs_ei, file.path(out_dir, "MVS_expected_influence_rank_residualized.csv"), row.names = FALSE)

```

10. Bridge expected influence computed manually

```
support_row_indices <- 14:25
mvs_col_indices     <- 1:13

W_support_to_mvs <- W[support_row_indices, mvs_col_indices, drop = FALSE]
BEI_manual <- rowSums(W_support_to_mvs)

support_node_names <- colnames(W)[support_row_indices]
source_group <- rep(c("Teacher", "Family", "Friend"), each = 4)

bei_manual_df <- data.frame(
  node = support_node_names,
  source = source_group,
  BEI = round(BEI_manual, 4)
)

mean_bei_manual <- aggregate(BEI ~ source, data = bei_manual_df, FUN = mean)
print("=== Mean bridge expected influence by support source ===")
print(mean_bei_manual)

write.csv(bei_manual_df, file.path(out_dir, "BEI_each_support_item_manual_residualized.csv"), row.names = FALSE)
write.csv(mean_bei_manual, file.path(out_dir, "mean_BEI_by_source_manual_residualized.csv"), row.names = FALSE)

```

11. Bootstrap comparison of mean BEI across support sources

```
boot_compare_BEI_manual <- function(data_list, nBoots = 1000, nCores = 4) {
  support_rows <- 14:25
  mvs_cols <- 1:13
  source_labels <- rep(c("Teacher", "Family", "Friend"), each = 4)

  compute_avg_BEI_once <- function(boot_sample) {
    R_boot <- qgraph::cor_auto(boot_sample)
    R_boot <- (R_boot + t(R_boot)) / 2   # numerical symmetrization: cor_auto output may not be exactly symmetric
    if (!matrixcalc::is.positive.definite(R_boot)) {
      R_boot <- as.matrix(Matrix::nearPD(R_boot, corr = TRUE)$mat)
    }
    # Estimate the network with the same ggmModSelect settings as the main analysis
    fit_boot <- ggmModSelect_safe(S = R_boot, n = nrow(boot_sample), gamma = 0.25,
                                  nLambda = 100, lambda.min.ratio = 0.01,
                                  stepup = TRUE, stepdown = TRUE)
    W_boot <- get_W(fit_boot)
    colnames(W_boot) <- rownames(W_boot) <- colnames(data_list[[1]])

    W_sub <- W_boot[support_rows, mvs_cols, drop = FALSE]
    BEI_vec <- rowSums(W_sub)

    teacher_mean <- mean(BEI_vec[source_labels == "Teacher"])
    family_mean  <- mean(BEI_vec[source_labels == "Family"])
    friend_mean  <- mean(BEI_vec[source_labels == "Friend"])

    return(c(Teacher = teacher_mean, Family = family_mean, Friend = friend_mean))
  }

  data_orig <- data_list[[1]]
  N_local <- nrow(data_orig)

  cl <- makeCluster(min(nCores, detectCores() - 1))
  registerDoParallel(cl)

  boot_results <- foreach(iter = 1:nBoots, .combine = rbind,
                          .packages = c("qgraph", "Matrix", "matrixcalc"),
                          .export = c("support_rows", "mvs_cols", "source_labels",
                                      "ggmModSelect_safe", "get_W")) %dopar% {
                                        set.seed(20260805 + iter)
                                        idx <- sample(1:N_local, size = N_local, replace = TRUE)
                                        boot_sample <- data_orig[idx, ]
                                        compute_avg_BEI_once(boot_sample)
                                      }
  stopCluster(cl)

  boot_df <- as.data.frame(boot_results)
  colnames(boot_df) <- c("Teacher", "Family", "Friend")
  return(boot_df)
}

```

```
cat("\nRunning the bootstrap difference test (", N_BOOTS, " iterations)...\n")
boot_means <- boot_compare_BEI_manual(node_list_resid, nBoots = N_BOOTS, nCores = N_CORES)

write.csv(boot_means, file.path(out_dir, "bootstrapped_mean_BEI_residualized.csv"), row.names = FALSE)

diff_TF  <- boot_means$Teacher - boot_means$Family
diff_TFr <- boot_means$Teacher - boot_means$Friend
diff_FFr <- boot_means$Family  - boot_means$Friend

ci_TF  <- quantile(diff_TF,  probs = c(0.025, 0.975), na.rm = TRUE)
ci_TFr <- quantile(diff_TFr, probs = c(0.025, 0.975), na.rm = TRUE)
ci_FFr <- quantile(diff_FFr, probs = c(0.025, 0.975), na.rm = TRUE)

################################################################################
## Significance of the bootstrap BEI difference test.
## A confidence interval that contains 0 is treated as nonsignificant, so only
## intervals lying strictly on one side of 0 are flagged as significant.
################################################################################
ci_contains_zero <- function(lo, hi) (lo <= 0) & (hi >= 0)   # closed interval contains 0

diff_results <- data.frame(
  Comparison = c("Teacher - Family", "Teacher - Friend", "Family - Friend"),
  Mean_Diff  = c(mean(diff_TF, na.rm = TRUE), mean(diff_TFr, na.rm = TRUE), mean(diff_FFr, na.rm = TRUE)),
  CI_lower   = c(ci_TF[1], ci_TFr[1], ci_FFr[1]),
  CI_upper   = c(ci_TF[2], ci_TFr[2], ci_FFr[2]),
  # A confidence-interval endpoint equal to 0 counts as nonsignificant
  Significant = !c(ci_contains_zero(ci_TF[1],  ci_TF[2]),
                   ci_contains_zero(ci_TFr[1], ci_TFr[2]),
                   ci_contains_zero(ci_FFr[1], ci_FFr[2]))
)
write.csv(diff_results, file.path(out_dir, "BEI_bootstrap_difference_test_residualized.csv"), row.names = FALSE)

cat("\n=== Bootstrap difference test on the mean BEI across support sources ===\n")
print(diff_results)

# Density plot
boot_df_long <- boot_means %>%
  pivot_longer(cols = c(Teacher, Family, Friend), names_to = "Source", values_to = "BEI")

mean_vals <- mean_bei_manual$BEI
names(mean_vals) <- mean_bei_manual$source

p_dist <- ggplot(boot_df_long, aes(x = BEI, fill = Source)) +
  geom_density(alpha = 0.4) +
  geom_vline(xintercept = mean_vals["Teacher"], color = "#E58606", linetype = "dashed", linewidth = 1) +
  geom_vline(xintercept = mean_vals["Family"],  color = "#54A24B", linetype = "dashed", linewidth = 1) +
  geom_vline(xintercept = mean_vals["Friend"],  color = "#D62728", linetype = "dashed", linewidth = 1) +
  scale_fill_manual(values = c(Teacher = "#E58606", Family = "#54A24B", Friend = "#D62728")) +
  theme_minimal() +
  labs(title = "Bootstrap Distribution of Mean BEI (Residualized)",
       x = "Mean Bridge Expected Influence (to MVS)", y = "Density")
ggsave(file.path(out_dir, "Figure_BEI_bootstrap_distribution_residualized.png"), p_dist, width = 10, height = 6, dpi = 300)
ggsave(file.path(out_dir, "Figure_BEI_bootstrap_distribution_residualized.pdf"), p_dist, width = 10, height = 6)

```

12. Bootstrap edge-weight CIs and centrality stability

```
df_boot <- node_list_resid[[1]]

# Network used as the basis for the bootstrap procedures
net_base <- bootnet::estimateNetwork(
  df_boot,
  default = "ggmModSelect",
  corMethod = "cor_auto",
  tuning = 0.25            # tuning is passed to ggmModSelect as gamma (0.25)
)
saveRDS(net_base, file = file.path(out_dir, "net_base_ggmModSelect_residualized.rds"))

# Nonparametric bootstrap: edge-weight confidence intervals
set.seed(20260805)
boot_edge <- bootnet::bootnet(
  net_base,
  nBoots = N_BOOTS,
  type = "nonparametric",
  statistics = c("edge", "expectedInfluence", "strength", "closeness", "betweenness"),
```

```
  nCores = N_CORES,
  parallel = "multicore"
)
saveRDS(boot_edge, file = file.path(out_dir, "boot_nonparametric_edges_residualized.rds"))

pdf(file.path(out_dir, "Figure_edge_CI_residualized.pdf"), width = 12, height = 8)
plot(boot_edge, labels = FALSE, order = "sample")
dev.off()

png(file.path(out_dir, "Figure_edge_CI_residualized.png"), width = 2600, height = 1800, res = 300)
plot(boot_edge, labels = FALSE, order = "sample")
dev.off()

pdf(file.path(out_dir, "Figure_edge_difference_residualized.pdf"), width = 12, height = 10)
plot(boot_edge, statistics = "edge", plot = "difference",
     onlyNonZero = TRUE, order = "sample")
dev.off()

png(file.path(out_dir, "Figure_edge_difference_residualized.png"), width = 2600, height = 2000, res = 300)
plot(boot_edge, statistics = "edge", plot = "difference",
     onlyNonZero = TRUE, order = "sample")
dev.off()

# Case-dropping bootstrap: centrality stability
set.seed(20260805)
boot_case <- bootnet::bootnet(
  net_base,
  nBoots = N_BOOTS,
  type = "case",
  statistics = c("expectedInfluence", "strength", "closeness", "betweenness"),
  nCores = N_CORES,
  parallel = "multicore"
)
saveRDS(boot_case, file = file.path(out_dir, "boot_caseDropping_centrality_residualized.rds"))

cs_coef <- bootnet::corStability(boot_case)
writeLines(capture.output(cs_coef), con = file.path(out_dir, "CS_coefficients_residualized.txt"))

pdf(file.path(out_dir, "Figure_centrality_stability_residualized.pdf"), width = 10, height = 7)
plot(boot_case, statistics = c("expectedInfluence", "closeness", "betweenness"))
dev.off()

png(file.path(out_dir, "Figure_centrality_stability_residualized.png"), width = 2400, height = 1700, res = 300)
plot(boot_case, statistics = c("expectedInfluence", "closeness", "betweenness"))
dev.off()

```

13. Export centrality values

```
export_centrality_values <- function(Wmat, out_path_prefix) {
  Wmat <- as.matrix(Wmat)
  Wmat[is.na(Wmat)] <- 0
  diag(Wmat) <- 0
  Wmat <- (Wmat + t(Wmat)) / 2
  nodes <- colnames(Wmat)
  if (is.null(nodes)) nodes <- paste0("V", 1:ncol(Wmat))

  cent <- qgraph::centrality(Wmat)
  strength    <- cent$OutDegree
  closeness   <- cent$Closeness
  betweenness <- cent$Betweenness
  if (is.null(names(strength)))    names(strength)    <- nodes
  if (is.null(names(closeness)))   names(closeness)   <- nodes
  if (is.null(names(betweenness))) names(betweenness) <- nodes

  expectedInfluence <- rowSums(Wmat)
  names(expectedInfluence) <- rownames(Wmat)

  df_raw <- data.frame(
    node = nodes,
    expectedInfluence = expectedInfluence[nodes],
    strength = strength[nodes],
    closeness = closeness[nodes],
    betweenness = betweenness[nodes]
  )
```

```
  df_z <- df_raw
  df_z[, -1] <- scale(df_raw[, -1])

  readr::write_csv(df_raw, paste0(out_path_prefix, "_centrality_raw.csv"))
  readr::write_csv(df_z,   paste0(out_path_prefix, "_centrality_z_sorted.csv"))
  return(list(raw = df_raw, z = df_z))
}

cent_out <- export_centrality_values(W, file.path(out_dir, "RESID_25node"))

cent_z_sorted <- cent_out$z[order(cent_out$z$expectedInfluence), ]
write.csv(cent_z_sorted, file.path(out_dir, "Figure2_centrality_data_residualized.csv"), row.names = FALSE)

```

14. Standardized centrality plot

```
pdf(file.path(out_dir, "Figure_centrality_standardized_residualized.pdf"), width = 10, height = 6)
qgraph::centralityPlot(W,
                       include = c("ExpectedInfluence", "Closeness", "Betweenness"),
                       orderBy = "ExpectedInfluence", scale = "z-scores",
                       labels = colnames(W))
dev.off()

png(file.path(out_dir, "Figure_centrality_standardized_residualized.png"),
    width = 3000, height = 1800, res = 300)
qgraph::centralityPlot(W,
                       include = c("ExpectedInfluence", "Closeness", "Betweenness"),
                       orderBy = "ExpectedInfluence", scale = "z-scores",
                       labels = colnames(W))
dev.off()

```

15. Completion note

```
writeLines(
  c("Supplementary 25-node network analysis completed.",
    paste0("Output directory: ", out_dir),
    paste0("Bootstrap iterations: ", N_BOOTS, " | worker cores: ", N_CORES),
    "",
    "Main outputs:",
    "  Figure_network_residualized.png/pdf              residualized network",
    "  RESID_25node_centrality_raw.csv / _z_sorted.csv   centrality values",
    "  Figure2_centrality_data_residualized.csv          centrality plot data",
    "  expected_influence_rank_residualized.csv          expected influence",
    "  MVS_expected_influence_rank_residualized.csv      materialism item EI",
    "  BEI_each_support_item_manual_residualized.csv     item-level BEI",
    "  mean_BEI_by_source_manual_residualized.csv        mean BEI by source",
    "  BEI_bootstrap_difference_test_residualized.csv    BEI difference test",
    "  precision_matrix_diagnostics.csv                  precision-matrix check",
    "  final_precision_matrix_theta.rds                  final precision matrix",
    "  CS_coefficients_residualized.txt                  stability coefficients",
    "  Figure_*_residualized.png/pdf                     figures",
    "",
    "Note: the network is a conditional dependence structure adjusted for",
    "parental education (averaged), monthly household income, gender, age",
    "and family residence."),
  con = file.path(out_dir, "README_residualized_analysis.txt")
)

cat("\nSupplementary 25-node network analysis completed.\n")
cat("Output directory:", out_dir, "\n")
```
