# Supplementary material for: Associations between social support and central components of adolescent materialism: a network analysis
Source: Front Psychol. 2026 Oct 14;17:1925963. doi: 10.3389/fpsyg.2026.1925963 (PMC13713670; doi:10.3389/fpsyg.2026.1925963)
Supplement: Supplementary file 2 [file Table_1.DOCX]

**Supplementary Table 1.** Descriptive statistics and normality diagnostics for MVS and PSSS items

| Node | *Mean* | *SD* | Skewness | Kurtosis |
| --- | --- | --- | --- | --- |
| MVS1 | 2.54 | 1.17 | .25 | −.99 |
| MVS2 | 2.61 | 1.07 | .33 | −.69 |
| MVS3 | 2.68 | .97 | .27 | −.21 |
| MVS4 | 3.25 | 1.09 | −.29 | −.68 |
| MVS5 | 2.67 | .91 | .26 | −.16 |
| MVS6 | 2.61 | .93 | .32 | −.08 |
| MVS7 | 3.27 | 1.03 | −.30 | −.43 |
| MVS8 | 2.77 | 1.07 | .19 | −.70 |
| MVS9 | 2.40 | 1.05 | .41 | −.60 |
| MVS10 | 3.28 | 1.03 | −.16 | −.39 |
| MVS11 | 3.12 | 1.16 | −.22 | −.81 |
| MVS12 | 2.92 | 1.16 | −.01 | −.84 |
| MVS13 | 2.29 | 1.07 | .60 | −.21 |
| Te1 | 4.02 | 1.43 | .06 | −.13 |
| Te2 | 3.61 | 1.55 | .25 | −.42 |
| Te5 | 3.70 | 1.58 | .07 | −.43 |
| Te10 | 3.71 | 1.59 | .03 | −.52 |
| Fa3 | 5.29 | 1.37 | −.61 | −.04 |
| Fa4 | 5.62 | 1.42 | −1.04 | .64 |
| Fa8 | 4.95 | 1.64 | −.60 | −.30 |
| Fa11 | 4.71 | 1.62 | −.26 | −.66 |
| Fr6 | 4.76 | 1.43 | −.34 | −.26 |
| Fr7 | 4.53 | 1.50 | −.30 | −.30 |
| Fr9 | 5.39 | 1.36 | −.82 | .51 |
| Fr12 | 5.21 | 1.38 | −.65 | .10 |

*Note.* MVS1 = “I admire people who own expensive homes, cars and clothes.” MVS2 = “I usually buy only the things I need.” MVS3 = “I put less emphasis on material things than most people I know.” MVS4 = “Some of the most important achievements in life include acquiring material possessions.” MVS5 = “I try to keep my life simple as far as possessions are concerned.” MVS6 = “I don’t place much emphasis on the amount of material objects people own as a sign of success.” MVS7 = “My life would be better if I owned certain things I don’t have.” MVS8 = “The things I own say a lot about how well I’m doing in life.” MVS9 = “I enjoy spending money on things that aren’t practical.” MVS10 = “I wouldn’t be any happier if I owned nicer things.” MVS11 = “Buying things gives me a lot of pleasure.” MVS12 = “I’d be happier if I could afford to buy more things.” MVS13 = “I like a lot of luxury in my life.” Te1 = “There is a teacher who is around when I am in need.” Te2 = “There is a teacher with whom I can share my joys and sorrows.” Fa3 = “My family really tries to help me.” Fa4 = “I get the emotional help and support I need from my family.” Te5 = “I have a teacher who is a real source of comfort to me.” Fr6 = “My friends really try to help me.” Fr7 = “I can count on my friends when things go wrong.” Fa8 = “I can talk about my problems with my family.” Fr9 = “I have friends with whom I can share my joys and sorrows.” Te10 = “There is a teacher in my life who cares about my feelings.” Fa11 = “My family is willing to help me make decisions.” Fr12 = “I can talk about my problems with my friends.”

**Supplementary Table 2.** Edge-weight matrix for MVS and PSSS items

|  | MVS1 | MVS2 | MVS3 | MVS4 | MVS5 | MVS6 | MVS7 | MVS8 | MVS9 | MVS10 | MVS11 | MVS12 | MVS13 | Te1 | Te2 | Te5 | Te10 | Fa3 | Fa4 | Fa8 | Fa11 | Fr6 | Fr7 | Fr9 | Fr12 |
| --- | --- | --- | --- | --- | --- | --- | --- | --- | --- | --- | --- | --- | --- | --- | --- | --- | --- | --- | --- | --- | --- | --- | --- | --- | --- |
| MVS1 | 0 |  |  |  |  |  |  |  |  |  |  |  |  |  |  |  |  |  |  |  |  |  |  |  |  |
| MVS2 | 0 | 0 |  |  |  |  |  |  |  |  |  |  |  |  |  |  |  |  |  |  |  |  |  |  |  |
| MVS3 | .17 | .22 | 0 |  |  |  |  |  |  |  |  |  |  |  |  |  |  |  |  |  |  |  |  |  |  |
| MVS4 | .18 | −.08 | 0 | 0 |  |  |  |  |  |  |  |  |  |  |  |  |  |  |  |  |  |  |  |  |  |
| MVS5 | 0 | .23 | .18 | 0 | 0 |  |  |  |  |  |  |  |  |  |  |  |  |  |  |  |  |  |  |  |  |
| MVS6 | 0 | 0 | .21 | 0 | .21 | 0 |  |  |  |  |  |  |  |  |  |  |  |  |  |  |  |  |  |  |  |
| MVS7 | .17 | −.08 | 0 | 0 | 0 | −.07 | 0 |  |  |  |  |  |  |  |  |  |  |  |  |  |  |  |  |  |  |
| MVS8 | 0 | 0 | 0 | .22 | 0 | .15 | .25 | 0 |  |  |  |  |  |  |  |  |  |  |  |  |  |  |  |  |  |
| MVS9 | 0 | .33 | 0 | 0 | 0 | 0 | 0 | .14 | 0 |  |  |  |  |  |  |  |  |  |  |  |  |  |  |  |  |
| MVS10 | 0 | 0 | 0 | 0 | 0 | .14 | 0 | 0 | −.13 | 0 |  |  |  |  |  |  |  |  |  |  |  |  |  |  |  |
| MVS11 | 0 | 0 | 0 | 0 | 0 | 0 | 0 | 0 | .15 | 0 | 0 |  |  |  |  |  |  |  |  |  |  |  |  |  |  |
| MVS12 | .13 | 0 | 0 | .11 | 0 | 0 | .29 | .14 | 0 | .08 | .33 | 0 |  |  |  |  |  |  |  |  |  |  |  |  |  |
| MVS13 | .15 | 0 | 0 | .10 | .14 | .09 | 0 | 0 | .15 | 0 | .16 | .28 | 0 |  |  |  |  |  |  |  |  |  |  |  |  |
| Te1 | 0 | 0 | 0 | 0 | 0 | 0 | 0 | 0 | 0 | 0 | 0 | 0 | 0 | 0 |  |  |  |  |  |  |  |  |  |  |  |
| Te2 | 0 | 0 | 0 | 0 | 0 | 0 | 0 | 0 | −.07 | 0 | 0 | 0 | 0 | .31 | 0 |  |  |  |  |  |  |  |  |  |  |
| Te5 | 0 | 0 | 0 | 0 | 0 | 0 | 0 | 0 | 0 | 0 | 0 | −.06 | 0 | .22 | .28 | 0 |  |  |  |  |  |  |  |  |  |
| Te10 | 0 | 0 | −.08 | 0 | 0 | 0 | 0 | 0 | 0 | 0 | 0 | 0 | 0 | .18 | .18 | .30 | 0 |  |  |  |  |  |  |  |  |
| Fa3 | 0 | 0 | 0 | 0 | 0 | 0 | 0 | 0 | 0 | 0 | 0 | 0 | 0 | 0 | .10 | 0 | 0 | 0 |  |  |  |  |  |  |  |
| Fa4 | 0 | 0 | 0 | 0 | 0 | 0 | 0 | 0 | 0 | 0 | 0 | 0 | 0 | 0 | 0 | .09 | 0 | .43 | 0 |  |  |  |  |  |  |
| Fa8 | 0 | 0 | 0 | 0 | 0 | 0 | 0 | 0 | −.09 | 0 | 0 | 0 | 0 | 0 | 0 | .09 | 0 | .14 | .32 | 0 |  |  |  |  |  |
| Fa11 | 0 | 0 | 0 | 0 | 0 | 0 | 0 | 0 | 0 | 0 | 0 | 0 | 0 | 0 | 0 | 0 | .09 | .09 | .08 | .13 | 0 |  |  |  |  |
| Fr6 | 0 | 0 | 0 | 0 | 0 | 0 | 0 | 0 | 0 | 0 | 0 | 0 | 0 | 0 | 0 | .07 | 0 | .07 | 0 | 0 | 0 | 0 |  |  |  |
| Fr7 | 0 | 0 | 0 | 0 | 0 | 0 | 0 | 0 | 0 | 0 | .06 | 0 | 0 | 0 | 0 | 0 | 0 | 0 | 0 | 0 | 0 | .52 | 0 |  |  |
| Fr9 | 0 | 0 | 0 | 0 | 0 | −.05 | 0 | 0 | 0 | 0 | 0 | 0 | 0 | 0 | 0 | 0 | .09 | 0 | 0 | .10 | 0 | .22 | .15 | 0 |  |
| Fr12 | 0 | 0 | 0 | 0 | 0 | 0 | 0 | 0 | 0 | 0 | 0 | 0 | 0 | 0 | 0 | 0 | 0 | 0 | 0 | 0 | .09 | .14 | .11 | .46 | 0 |

*Note.* MVS1 = “I admire people who own expensive homes, cars and clothes.” MVS2 = “I usually buy only the things I need.” MVS3 = “I put less emphasis on material things than most people I know.” MVS4 = “Some of the most important achievements in life include acquiring material possessions.” MVS5 = “I try to keep my life simple as far as possessions are concerned.” MVS6 = “I don’t place much emphasis on the amount of material objects people own as a sign of success.” MVS7 = “My life would be better if I owned certain things I don’t have.” MVS8 = “The things I own say a lot about how well I’m doing in life.” MVS9 = “I enjoy spending money on things that aren’t practical.” MVS10 = “I wouldn’t be any happier if I owned nicer things.” MVS11 = “Buying things gives me a lot of pleasure.” MVS12 = “I’d be happier if I could afford to buy more things.” MVS13 = “I like a lot of luxury in my life.” Te1 = “There is a teacher who is around when I am in need.” Te2 = “There is a teacher with whom I can share my joys and sorrows.” Fa3 = “My family really tries to help me.” Fa4 = “I get the emotional help and support I need from my family.” Te5 = “I have a teacher who is a real source of comfort to me.” Fr6 = “My friends really try to help me.” Fr7 = “I can count on my friends when things go wrong.” Fa8 = “I can talk about my problems with my family.” Fr9 = “I have friends with whom I can share my joys and sorrows.” Te10 = “There is a teacher in my life who cares about my feelings.” Fa11 = “My family is willing to help me make decisions.” Fr12 = “I can talk about my problems with my friends.”
